# The relationship between patient physiology and cancer-specific survival following curative resection of colorectal cancer

**DOI:** 10.1038/sj.bjc.6603560

**Published:** 2007-01-23

**Authors:** J T Jenkins, G O'Neill, C G Morran

**Affiliations:** 1 Department of Surgery, Crosshouse Hospital, Kilmarnock, UK

**Keywords:** POSSUM, colon, rectum, survival, Physiology Score

## Abstract

The impact of patient physiology on cancer-specific survival is poorly documented. Patient physiology predicted overall, cancer-specific (Physiology Score*>*30; HR 8.64 (95% CI 3.00–24.92); *P*=0.0005) and recurrence-free survival (Physiology Score *>*30; HR 7.44 (95% CI 1.99–27.73); *P*=0.003) independent of Dukes stage following potentially curative surgery for colorectal cancer. This independent negative association with survival is a novel observation.

Several factors have independent prognostic significance to cancer-specific survival following colorectal cancer (CRC) resection that may be classified according to tumour stage, clinical, pathologic, oncogenetic, molecular and immunologic variables (Chapuis *et al*, 1985; Hole and McArdle, 2002; McArdle and Hole, 2002). Recently, the systemic inflammatory response in CRC survival, as determined by C-reactive protein (CRP), has provoked interest (Shunyakov *et al*, 2004) and may reflect a tumour's malignant potential. Elevated CRP levels have been found to predict survival, independent of Dukes stage (McMillan *et al*, 2003; Canna *et al*, 2004), although others refute this finding (Chung and Chang, 2003a, 2003b). The systemic inflammatory response may also predict first cardiac events (Lloyd-Jones and Levy, 2003) and diabetes onset (Pradhan *et al*, 2001, 2003). Obesity and smoking are associated with the systemic inflammatory response (Ohsawa *et al*, 2005; Park *et al*, 2005). These comorbidities are likely to affect patient physiology. Whether these factors combine with oncological factors to impair survival is unclear. Few colorectal studies incorporate comorbidities in their predictive models of cancer-specific and overall survival. Canna *et al* (2004) combined CRP and Dukes stage to provide a more accurate assessment of prognosis, with promising results, however, few data exist that assess patient physiology and cancer outcomes.

For surgical patients, the accurate staging and prediction of peri-operative complications are fundamental to treatment planning. Accurate evaluation of physiological derangement requires comparatively few parameters. The Physiological and Operative Severity Score for the enumeration of Mortality and Morbidity (POSSUM) was developed in 1991 (Copeland *et al*, 1991) from multivariate discriminant analysis, and uses 12 independent physiological variables to provide a Physiology Score and a six-factor Operative Severity Score, for predicting operative mortality. Further refinements have generated the Colorectal (CR) POSSUM (Tekkis *et al*, 2004), where six of the 12 original factors were independent predictors of operative outcome for colorectal surgery.

The aim of this study was to examine the relationship between patient physiology and overall, recurrence-free and cancer-specific survival after potentially curative resection of CRC.

## METHODS

A prospective cohort study was conducted based on consecutive patients undergoing colon and rectal cancer resection from January 1996 to January 2001, followed until death or to June 2005 with data recorded prospectively in a computerised database.

Operations were classified as elective, emergency, urgent and scheduled. Preoperative physiology was assessed using POSSUM Physiology Score and categorised into four groups: 11–14, 15–20, 21–30 and >30 (Tekkis *et al*, 2004). Histopathological examination of all surgical specimens followed a standard protocol. Dukes (Dukes and Bussey, 1958) and TNM systems were used. Tumour differentiation was classified as well moderate and poor (Halvorsen and Seim, 1988). Only potentially curative resections were considered in this study. Palliative resections were defined as those with known residual tumour, either distant or local. Cause of death was recorded prospectively by colorectal nurse specialists, consultant surgeons and the Clinical Effectiveness Unit of the hospital and the mortality coding protocol for the study categorised death as CRC-related or from other causes/non-CRC-related. If post-mortem examination was performed then this information was considered.

### Statistical analyses

Overall survival analysis evaluated deaths from all causes. Observations were uncensored (complete) if the patient died of any cause in the follow-up period. This included postoperative deaths, deaths from CRC and deaths from events unrelated to CRC.

Cancer-specific survival analysis evaluated deaths from CRC. Observations were uncensored (complete) only if the patient died as a direct result of CRC in the follow-up period. Cancer-specific survival time was measured from the date of resection to the date of death owing to CRC, with patients lost to follow-up and those who died of other causes censored in survival analysis.

Recurrence-free survival related to the identification of recurrent CRC based upon clinical, pathological or radiological findings. Observations were uncensored (complete) if a patient was identified as having recurrent disease, locally or systemically, during the follow-up period.

Comparisons of survival time between strata of categorical variables were made using the Kaplan–Meier method (Kaplan and Meier, 1958) and log-rank test (Wellek, 1993). In addition, survival analyses employed the Cox proportional hazards model for variables assessed for prognosis. Multiple regression for variables showing a significant association with survival employed the Cox (1972) method. Statistical significance was set at 0.05 and confidence intervals (CI) were 95%. *SPSS for Windows Version 13.0* was used for statistical analyses.

## RESULTS

Five hundred and thirty-eight CRC resections were performed over the 5 year period to 2001. Four hundred and thirty-two resections were with curative intent and this study only considers survival in this group of patients. One patient was unspecified in surgical intent. Follow-up data were absent in five patients who were censored in analysis of overall, recurrence-free and cancer-specific survival. At the end of the study period, 226 (52.3%) were dead and 206 (47.7%) were alive. Sixty-three (14.6%) patients succumbed to CRC, and the median survival in this group was 22 months (IQR 13–40). One hundred and sixty-three patients (37.7%) died from other causes. The median follow-up for survivors was 74 months (IQR 60.5–88.0). Summary data are presented in Table 1.

In the curative group, neo-adjuvant or adjuvant therapies were given in 96 (22.2%) patients. Sixty-eight received chemotherapy; 11 combined postoperative chemoradiotherapy; four combined preoperative chemoradiotherapy; six preoperative radiotherapy; three postoperative radiotherapy; four received ‘other’ treatments.

Of the comorbidities assessed, 99 patients were smokers (22.9%), 129 were ex-smokers (29.9%) and 202 non-smokers (46.8%). Data were unrecorded in two patients. Seventy-eight patients had COPD/asthma (18.1%), 22 had a previous myocardial infarction (5.1%) with 111 documented with ischaemic heart disease (IHD) (25.7%), hypertension was documented in 134 (31.0%), diabetes in 28 (6.5%) and a ‘renal’ history in nine (2.1%). one hundred and sixty six patients had two or more comorbidities (38.4%).

**Figure 1:**
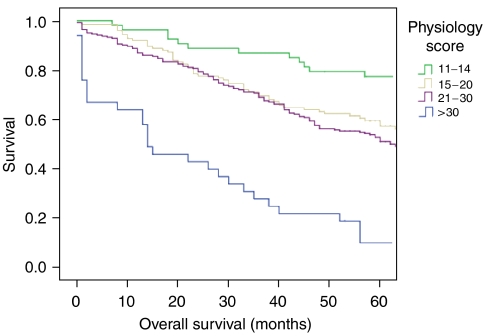
Five-year overall survival curve according to patient physiology after curative resection of 432 colorectal cancers.

**Figure 2:**
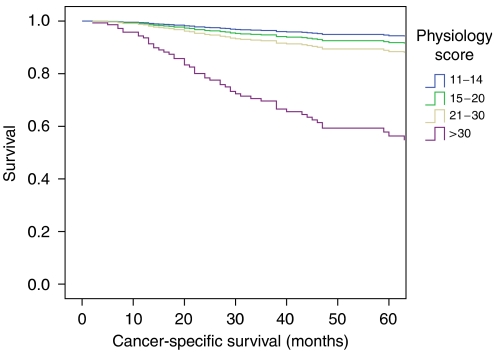
Five-year cancer-specific survival curve according to patient physiology after curative resection of 432 colorectal cancer patients.

**Figure 3:**
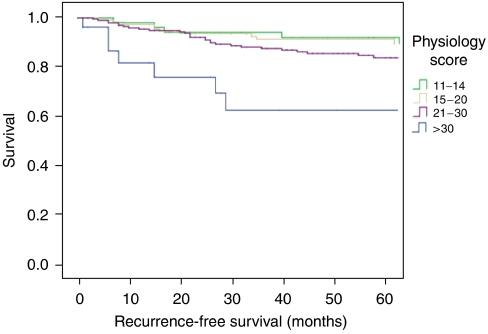
Five-year recurrence-free survival curve according to patient physiology after curative resection of 432 colorectal cancer patients.

Overall survival in relation to patient physiology is shown in Figure 1, and a significant association with overall survival is identified (log rank 79.24, df=3, *P*=0.0005). The effect of patient physiology on 5-year cancer-specific survival and 5-year recurrence-free survival are considered in Figures 2 and 3, respectively, where significant associations are also identified.

Univariate survival analyses for curative resections found Dukes stage, tumour differentiation, adjuvant therapy use and Physiology Score to significantly predict cancer-specific (Table 2) and recurrence-free survival (Table 3). Postoperative complications were significantly related to recurrence-free survival.

Multivariate analysis found patient physiology (*P*=0.0005) and Dukes stage (*P*=0.0005) to be independent predictors of cancer-specific survival (Table 2) and patient physiology, Dukes stage and postoperative complications to be significant for recurrence-free survival (Table 3).

In the group of patients at high risk of developing tumour recurrence, that is, Dukes C, we noted a significant association with patient physiology and the likelihood of adjuvant/neo-adjuvant therapy being administered (*χ*^2^=35.23, df=3; *P*=0.0005). Of the 155 Dukes C patients, 77 (49.3%) received adjuvant therapies and 78 (50.7%) received no additional therapy. When patients were categorised by scores of <30 or >30, patients in the better physiology group were significantly more likely to receive adjuvant therapy (odds ratio (OR)=5.36 (95% CI 1.64–17.59); *P*=0.002). When adjuvant/neo-adjuvant therapy is subdivided by Physiology Scores we find: 11–14; 20 adjuvant therapy, two no adjuvant therapy; 15–20; 29 adjuvant therapy, 14 no adjuvant therapy; 21–30; 27 adjuvant therapy, 52 no adjuvant therapy; >30; one adjuvant therapy, 10 no adjuvant therapy. The numbers of recurrences in each group were 11–14: five out of 22: 15–20; 10 out of 43; 21–30: 23 of 79; >30: eight out of 11. In the group which received adjuvant/neo-adjuvant treatment, patient physiology was not significantly associated with recurrence (*χ*^2^=3.26, *df*=3; *P*=0.353). However, in the group receiving no adjuvant/neo-adjuvant therapy, a significant association with recurrence and poorer physiology was identified (*χ*^2^=8.743.26, *df*=3; *P*=0.033).

## DISCUSSION

In the present study, we examined the influence of patient physiology on cancer-specific and recurrence-free survival in a group of consecutive patients assessed after potentially curative CRC resection from a west of Scotland population. The study has prospectively pursued a large number of cases with an almost complete dataset and accurate follow-up in over 98% of patients.

On univariate and multivariate analyses, patient physiology, as assessed by the POSSUM Physiology Score and Dukes stage were independent predictors of cancer-specific and recurrence-free survival. Currently, no evidence is available to make valid comparisons of cancer outcomes in relation to physiology.

The mechanism by which deranged patient physiology may influence cancer survival is not clear, and no clear explanations are provided from this study. We have based the physiology score categories on previously published data (Tekkis *et al*, 2004). However, we note that we have documented a higher proportion of cases with elevated or poorer physiology scores than the cited study, for example, 21–30 (51 *vs* 15%) and >30 (8 *vs* 3%). These differences may highlight geographical variations in general health, and the physiology findings may, therefore, not be applicable to the UK population on the whole. Issues specifically concerning deprivation have not been assessed in this study. Moreover, the difference in proportions cannot be explained by differences in the proportion of emergency procedures as the 5.1% rate observed is in keeping with UK national data.

Of interest, we noted that patients with better physiology were significantly more likely to receive adjuvant therapy (OR 5.36 (95% CI 1.64–17.59); *P*=0.002) for Dukes C lesions. It may be disputed that if patients with comorbid illness and poor physiology were to develop a local or distant recurrence, they may be denied further treatments including chemotherapy, radiotherapy or salvage surgery, as they may be too unfit. Alternatively, patients with a good physiology score and tumour recurrence may have a better prognosis and survival if their recurrence is treated aggressively. If this were the case then our results would be distorted. However, in the group of patients who received no adjuvant/neo-adjuvant therapy, those with poorer physiology were still significantly more likely to have recurrent disease than those with better physiology. Moreover, in multivariate analysis, the use of adjuvant/neo-adjuvant therapy was not found to be an independent predictor of survival, providing less emphasis to the caveat above.

We noted that postoperative complications were independently associated with recurrence-free survival, but not cancer-specific survival. All complications were considered together (anastomotic leaks *n*=8; respiratory tract infections *n*=59 (13.7%); cardiac complications *n*=47 (10.9%); renal complications *n*=20 (4.6%); thromboembolic events *n*=10 (2.3%); haemorrhage *n*=4 (0.9%); wound infection *n*=48 (11.1%)). The impact of anastomotic leak on immediate postoperative mortality and local recurrence is well recognised (Bell *et al*, 2003; Walker *et al*, 2004). Recent reports highlight importance in relation to long-term overall and cancer-specific survival (McArdle and McMillan, 2005), although most previous studies have had numbers of anastomotic leaks that are too small to allow the detection of differences in survival. The small numbers of clinical anastomotic leaks (*n*=8) in this cohort has precluded meaningful survival analysis. Other groups have found postoperative infective complications to be independently associated with cancer outcomes with inferior survival in those sustaining complications (Nespoli *et al*, 2004). Poorer recurrence-free survival may result from delays in the initiation of adjuvant therapy or avoidance owing to prolonged patient recovery or ill health. An alternative hypothesis is that postoperative complications may modify the systemic inflammatory response and provide favourable conditions for tumour growth and hence recurrence.

In summary, disturbed patient physiology contributes to poorer cancer-specific and recurrence-free survival of patients who have undergone potentially curative surgery for CRC. This represents a novel observation. Survival estimates may be enhanced by the addition of clinical characteristics, for instance, patient physiology to existing prognostic classifications.

## Figures and Tables

**Table 1:** Characteristics of 432 patients who underwent curative resection of colorectal cancer

**Patients**	***n*=432**	**%**
*Median age (IQR)*	72 (63–78)	—
*Gender*	221M : 211F	
Elective	344	79.6
Emergency	23	5.3
Urgent	22	5.1
Scheduled	43	10.0
		
*Physiology Score*		
11–14	53	12.3
15–20	124	28.8
20–30	220	51.2
>30	33	7.7
		
*Tumour differentiation*		
Well	72	16.7
Intermediate	278	64.4
Poor	66	15.3
Unrecorded	16	3.7
		
*Dukes stage*		
Dukes A	105	24.3
Dukes B	172	39.8
Dukes C	155	35.9
Dukes D	—	—
		
*All additional therapies*		
Yes	96	22.2
No	336	77.8
		
*Disease recurrence*		
Yes	63	14.6
No	370	85.4
		
*Cause of death*		
CRC related	63	14.6
Other cause/non-CRC related	163	37.7

CRC=colorectal cancer; IQR=interquartile range.

**Table 2:** Relationship between variables and cancer-specific survival following curative resection for colorectal cancer of 432 patients

	**Univariate analysis**		**Multivariate analysis**	
**Variable**	**Hazard ratio (95%CI)**	***P*-value**	**Hazard ratio (95%CI)**	***P*-value**
Age (<65/65–74/>75 years)	1.71 (0.87–3.37)	0.12	—	—
Gender (male/female)	1.11 (0.68–1.82)	0.69	—	—
NCEPOD classification (elective/emergency/urgent/scheduled)	**0.03 (1.29–7.09)**	**0.011**	1.24 (0.69–2.24)	0.466
Physiology Score (11–14/15–20/21–30/>30)	**7.38 (2.70–20.19)**	**0.0005**	**8.64 (3.00–24.92)**	**0.0005**
Dukes stage (Dukes A/B/C)	**14.68 (4.56–47.26)**	**0.0005**	**15.02 (4.65–48.48)**	**0.0005**
Tumour differentiation (well/moderate/poor)	**3.65 (1.43–9.32)**	**0.007**	1.52 (0.57–4.08)	0.40
Tumour site (colon/rectum)	0.85 (0.36–2.01)	0.71	—	—
All adjuvant therapies (yes/no)	**1.96 (1.17–3.27)**	**0.01**	0.93 (0.50–1.74)	0.83
Smoking history (yes/no)	0.98 (0.60–1.61)	0.94	—	—
Postoperative complications (yes/no)	1.61 (0.96–2.68)	0.07	—	

CI=confidence interval; NCEPOD=National Confidential Enquiry into Perioperative Deaths.

Bold values signify *P*<0.05.

**Table 3:** Relationship between variables and recurrence-free survival following curative resection for colorectal cancer of 432 patients

	**Univariate analysis**		**Multivariate analysis**	
**Variable**	**Hazard ratio (95% CI)**	***P*-value**	**Hazard ratio (95% CI)**	***P*-value**
Age (<65/65–74/>75 years)	**2.46 (1.20–5.07)**	**0.015**	2.12 (0.80–5.61)	0.129
Gender (male/female)	0.99 (0.59–1.70)	0.995		
NCEPOD classification (elective/emergency/urgent/scheduled)	1.85 (0.89–3.80)	0.311		
Physiology Score (11–14/15–20/21–30/>30)	**8.27 (2.85- 24.01)**	**0.0005**	**7.44 (1.99–27.73)**	**0.003**
Dukes stage (Dukes A/B/C)	**35.60 (5.30–280.77)**	**0.0005**	**29.94 (3.84–233.24)**	**0.001**
Tumour differentiation (well/moderate/poor)	**7.30 (2.12–25.05)**	**0.002**	2.77 (0.78–9.89)	0.116
Tumour site (colon/rectum)	1.09 (0.422–2.81)	0.861		
All adjuvant therapies (yes/no)	**1.81 (1.03–3.19)**	**0.04**	1.24 (0.58–2.65)	0.585
Smoking history (yes/no)	0.95 (0.55–1.61)	0.838		
Postoperative complications (yes/no)	**1.72 (1.01–2.96)**	**0.05**	**1.82 (1.03–3.22)**	**0.04**

CI=confidence interval; NCEPOD=National Confidential Enquiry into Perioperative Deaths.

Bold values signify *P*<0.05.
